# Acute Abdomen due to Primary Omental Torsion and Infarction

**DOI:** 10.1155/2014/208382

**Published:** 2014-11-06

**Authors:** S. Occhionorelli, M. Zese, L. Cappellari, R. Stano, G. Vasquez

**Affiliations:** ^1^Department of Morphology, Surgery and Experimental Medicine, University of Ferrara, Via L. Borsari 46, 44121 Ferrara, Italy; ^2^Department of Surgery, Emergency Surgery Service, Arcispedale Sant'Anna, Via A. Moro 8, 44124 Ferrara, Italy

## Abstract

*Background*. Torsion of greater omentum is a quite uncommon cause of acute abdomen. It can be primary or secondary but in both cases omentum twists upon itself and causes omental segmentary or diffuse necrosis. Symptoms are unspecific and preoperative diagnosis is difficult. The widespread and increasing use of computer tomography (CT) in differential diagnosis of acute abdomen can be useful for making a specific diagnosis. *Objectives*. This work aims to describe primary omental torsion in order to help avoid misdiagnosis, especially with acute appendicitis, which is eventually based solely on a physical examination. *Case Report*. We present a case of primary omental torsion in a young man and discuss contemporary methods in diagnosis and management of the condition. *Conclusions*. When a right diagnosis has been posed, possible treatments for omental torsion and necrosis are two: conservative or surgical. Conservative treatment had been rarely carried out because of frequent and important sequelae just like abdominal abscesses. Nowadays, surgical treatment, laparoscopic or laparotomic, is preferred because it is a safe method in diagnosis and management of this condition.

## 1. Introduction

Torsion of the greater omentum can be either primary or secondary. Primary torsion of the greater omentum, first reported by Eitel in 1899, occurs when the omentum twists upon itself, with the formation of a narrow neck in the absence of associated intra-abdominal pathology [1, 2]. Since then, there have been over 250 reported cases in the world literature [3, 4]. It mainly affects adults, with men being involved twice as frequently as women, with the majority being overweight [5]. It is quite difficult to establish a preoperative diagnosis of the condition [6, 7], but with wide use of computed tomography (CT) in patients with acute abdomen, this rare disease may be accurately diagnosed before surgery [2]. We report a case of primary omental torsion in a young man and discuss contemporary methods in diagnosis and management of the condition.

## 2. Case Report

**Figure 1:**
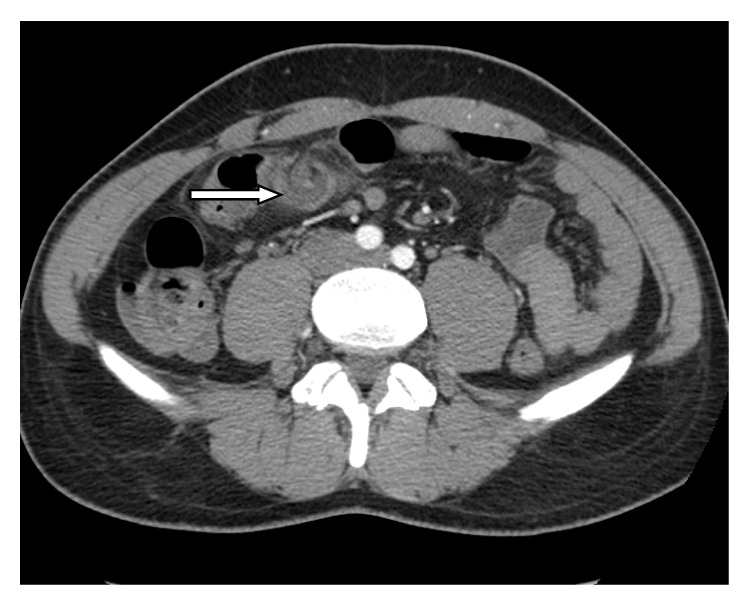
Axial contrast-enhanced CT scans obtained at the twisted point of omentum.

**Figure 2:**
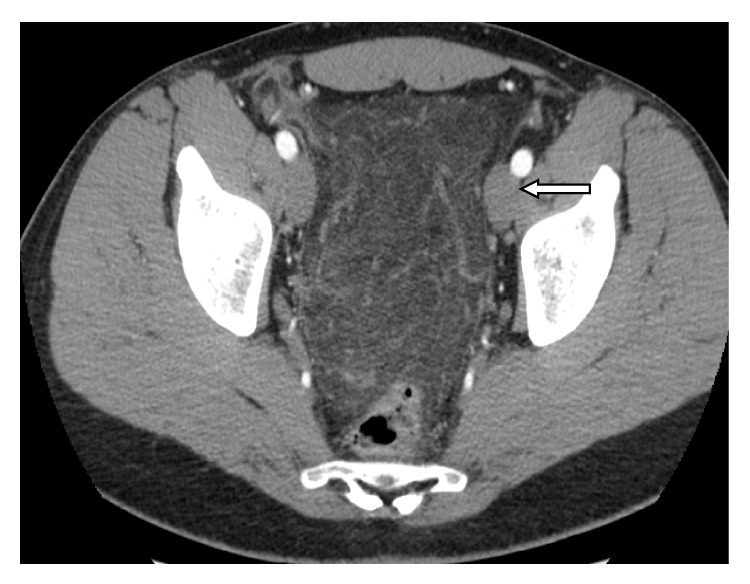
Axial contrast-enhanced CT scans obtained at the pelvis.

A twenty-nine-year old man was admitted to the Emergency Surgery Department of Sant'Anna University Hospital with a three-day history of epigastric and right-sided abdominal pain that was increased in severity, associated with nausea, vomiting, and anorexia. In his past history, only an episode of acute appendicitis occurred 3 months before and it was treated conservatively. On physical examination, the patient had a pulse of 75 beats/min, blood pressure of 110/60 mmHg, and a temperature of 37.2°C. Abdominal examination revealed tenderness and guarding especially in the right abdomen with diminished abdomen sounds. McBurney sign was positive but all the other appendicular signs were negative. No masses were palpable. Lungs were clean to auscultation and the cardiocirculatory examination was negative. Laboratory tests noticed leukocytosis (neutrophils 10.3 × 10^3^/*μ*L) and CRP was 1.4 mg/dL. An ultrasound scan showed, in correspondence with the right paraumbilical region, an oval hyperechoic region bounded by a hypoechoic rib with a small fluid district. This finding was situated immediately behind the rear surface of the abdominal wall and was not of unique interpretation, possible for herniation of bowel loop or intussusception. An abdominal computed tomography (CT) scan (Figures 1 and 2) revealed twisting of the omentum with an aspect of multiple targets and fluid district. The fat tissue situated near there and in the pelvic cavity appears much denser than standard and processes to plausible venous stasis and inflammation.

**Figure 3:**
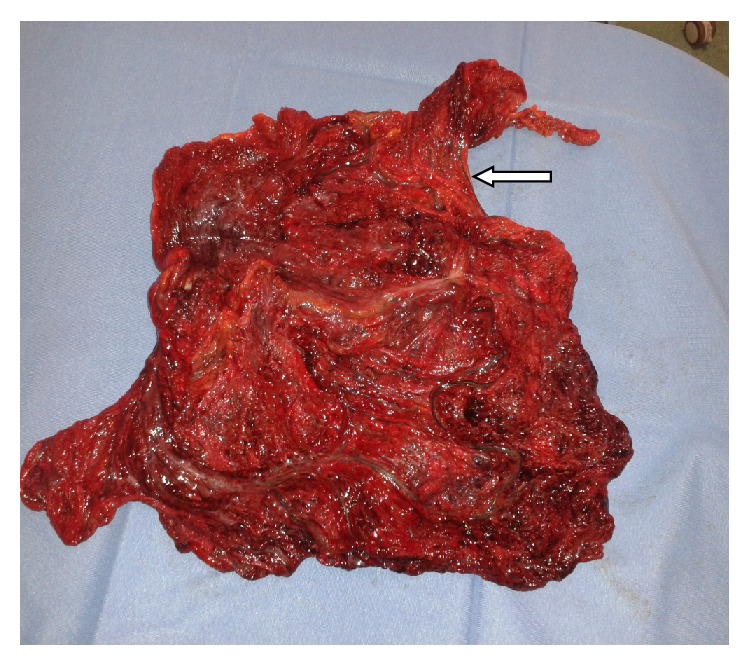
Twisted omentum.

The patient was observed with conservative management. After 12 hours, we noticed an increase in abdominal rebound tenderness and guarding. Laboratory tests showed a decreased leukocytosis (neutrophils 7.74 × 10^3^/*μ*L) but CRP was increased (8.4 mg/dL). Therefore, we decided to perform an explorative laparoscopy, which revealed a large necrotic area in the abdomen and a widespread hemorrhagic infarction. We subsequentially decided to perform a midline laparotomy. Omentum appeared widely necrotic, involving both the right and left sides, by a torsion that occurred at its superior point of attachment to the transverse colon (Figure 3). Hemorrhagic fluid was collected in the entire peritoneal cavity but bowel was not suffering. Appendix and gallbladder were normal; Meckel diverticulum was not present. We decided to perform a near total omentectomy and a prophylactic appendectomy. The postoperatory course was regular; the patient started to eat on the third postoperatory day and was discharged on the fifth postoperatory day in good clinical conditions.

## 3. Discussion

Omental infarction, with or without torsion, is a rare cause of acute abdominal pain, which makes it a difficult and unusual diagnosis to make. When compared with appendicitis, torsion has an incidence of 0.0016% to 0.37%, which is a ratio of less than 4 cases per 1000 cases of appendicitis [8, 9].

Omental torsion can present in 2 ways. In primary torsion, anatomic malformations such as a bifid or accessory omentum cause a spontaneous torsion; sudden movements, violent exercise, and hyperperistalsis have been implicated as precipitating factors. Obesity is also a well-documented risk linked to primary torsion, with one study documenting that almost 70% of patients with omental infarction were obese [10]. It is postulated that excess fat unevenly distributed in the omentum acts as a lead point for torsion. Secondary torsion occurs most often because of hernia, tumor, or adhesion, with the dependent omentum becoming fixed in the torsed position and unable to untwist. Both of these processes may lead to infarction of the affected omentum [11]. Our patient seems to have had none of these predisposing or precipitating conditions mentioned.

The primary symptom associated with omental torsion is pain, which is frequently localized in the right lower quadrant of the abdomen. The onset of pain is usually sudden and does not radiate to the abdominal wall [12]. In many cases, the pain localizes in the right lower quadrant and reveals signs of peritoneal irritation. Bowel movements are usually normal, and nausea and vomiting are rare. A thorough blood workup reveals normal values in many cases [4, 13].

The great majority of cases of omental torsion and infarction reported in the literature were segmental involving the right side of the omentum [14, 15]. Left-sided omental torsion is occasional but has been described [16]. Our case had diffuse infarction of both right and left sides of greater omentum with omental torsion located anteriorly just in front of traverse colon.

Differential diagnosis should include appendicitis, cholecystitis, cecal diverticulitis, perforated duodenal ulcer, abdominal wall hematoma, and intestinal obstruction [6, 17]. In women of reproductive age, salpingitis, ovarian cyst torsion, and ectopic pregnancy should also be considered [6]. In children, differential diagnosis should also include Meckel diverticulum and mesenteric adenitis [6]. Finally, torsion of accessory spleen is another diagnostic possibility, due to the fact that accessory spleen, when it exists, usually resides inside the omentum [18]. In our case, the diagnosis might have been of acute appendicitis if it were based only on the past history and physical examination, as reported in Alvarado Score [19–21]. Because the condition falls in the clinical context of acute abdomen, ultrasound (US) and CT scans are often performed to assist the diagnosis. US findings in omental torsion are usually consistent with a hyperechoic, noncompressible ovoid intra-abdominal mass adherent to the abdominal wall, which is located in the umbilical region or anterolaterally to the right half of the colon. US also eliminates acute cholecystitis [4, 14, 22, 23]. CT scan is considered the examination of choice in cases of acute abdomen [17]. If CT shows normal gallbladder and appendix with no signs suggestive of diverticulitis, the differential diagnosis is limited [7, 17]. Specific CT findings in omental torsion include diffuse streaking in a whirling pattern of fibrous and fatty folds [24]. A basic advantage of CT versus a US scan is the reliability of identifying the mass in the characteristic location between the anterior abdominal wall and the colon [25]. It has been reported that either nonoperative or preoperative diagnosis is only made in 0.6% to 4.8% of cases of omental infarction [11]. In our case, though, CT demonstrates the twisted omental part but US scan was not specific.

Two treatments are predominant: early laparoscopic surgical intervention and conservative medical treatment. Conservative treatment for omental infarction varies among physicians and includes all or part of the following: oral analgesics, anti-inflammatory drugs, and prophylactic antibiotics [26, 27]. Complications of conservative management include abscesses and adhesions induced by the persistence of necrotic tissue in the abdomen [11, 28–30]. More importantly, a missed diagnosis of acute appendicitis could have disastrous consequences [11]. In the literature, successful conservative treatment had been reported in only seven cases of segmental omental infarction that was eventually atrophied and/or fibrotic on radiologic follow-up [4]. Surgical resection of the affected omentum is usually the treatment of choice and laparoscopic surgery is an alternative treatment of choice [4, 29]. In our case the first approach was laparoscopic, but the important abdominal situation required a more secure laparotomic approach. Appendectomy was performed in a way to avoid a possible future intervention.

## 4. Conclusion

Omental torsion is a very rare condition, which can create problems in differential diagnosis of the acute abdomen especially with acute appendicitis. No one can rule out the fact that the previous episode of acute appendicitis accused by the patient three months earlier was actually a first symptom of a partial omental torsion and then resolved spontaneously; moreover, at the time of the intervention, the appendix was retrieved healthy. Approaching this kind of patients on the basis of the past history and physical examination only applying an Alvarado Score [19–21] can be dangerous and can lead to dramatic errors in diagnosis.

CT and ultrasound scans are very useful in order to diagnose a suspect omental torsion, and the right approach to the disease, in our opinion, is surely surgical. Laparoscopy has been decided to explore the entire abdomen cavity. A single classic iliac laparotomic access usually used for appendectomy in our case probably would give rise to a potentially fatal outcome, based on a dramatic diagnostic error.
